# Higher temperature and substrate vibrations as stress factors for terrestrial isopods – model species matter

**DOI:** 10.3897/zookeys.1101.77549

**Published:** 2022-05-18

**Authors:** Barbora Ďurajková, Richard Hladký, Ivan Hadrián Tuf

**Affiliations:** 1 Department of Ecology and Environmental Sciences, Faculty of Science, Palacky University Olomouc, Slechtitelu 27, CZ-77900 Olomouc, Czech Republic Palacky University Olomouc Czech Republic

**Keywords:** Aggregation, Isopoda, Oniscidea, stress factor, turn alternation

## Abstract

This study was focused on behaviour of the Common Rough Woodlouse (*Porcellioscaber*) and the Plum Woodlouse (*Porcellionidespruinosus*) under the influence of stressors in the form of increased temperature, the vibrating surface, or their combination. Two types of experiments were performed. First, woodlice placed in a labyrinth were observed, to determine the degree of turn alternation, the speed of passing through the labyrinth, and the corrections of turn alternation, when exposed to stressors. In the second experiment how woodlice aggregate in the aforementioned potential stressors was recorded and whether the change in aggregation behaviour can be an indicator of the degree of stress. Increased temperature and the combination of increased temperature and vibrations were stressors only for *P.scaber*. The results show that vibrations are not a stress factor for *P.scaber* or *P.pruinosus*. *Porcellioscaber* passed through the labyrinth more slowly at increased temperatures, and although they made more turn-corrections, they alternated turns less intensely. Its aggregation behaviour was mainly influenced by temperature, which confirms that the aggregation behaviour of *P.scaber* actually indicates a degree of stress.

## Introduction

Humans are not the only ones to face stress. Although this may seem trivial from today’s perspective, soil invertebrates such as isopods may also suffer from stress (Elwood et al. 2009). They have to solve existential problems to fulfil their necessities, such as the need for water or moisture (Cloudsley-Thompson 1956), food (Brody and Lawlor 1984), shelter (Allee 1926), and mating (Sutton 1972). This all takes place at constant risk of predation, either by one’s own kind (cannibalism) (Sutton 1972) or by other species such as spiders (Gorvett 1956), ants, birds, amphibians, and mammals (Hegarty and Kight 2014). If isopods suffer from a deficiency or excess of any of these factors or the risk of predation, their behaviour may be affected (Sutton 1972). Just like vertebrates, invertebrates respond to stress caused by changes in the environment with a stress reaction. Isopods secrete substances into the haemolymph which are similar to glucocorticoids that can be found in vertebrates (Elwood et al. 2009). Stress can also shorten the maternal care period of the clutch (Kight and Nevo 2004). Isopod response to specific stimuli in nature is influenced by the mutual effect of distinctly intense individual stimuli and also by the current physiological state of isopods (Sutton 1972).

In the present study, we examined two types of defence behaviour of isopods, namely systematic turn alternation and the formation of an aggregation. A tendency to alternate turns is a behaviour known for different organisms including humans. Turn alternations are characterised by two types of reactions. A spontaneous reaction (Richman et al. 1986) is based on environmental stimuli, such as rodents’ responses to a new maze (Montgomery 1952), or the reaction of cockroaches to a change in luminosity in a maze (Wilson and Fowler 1976). The second type of turn alternations is caused by the body’s internal physiological response (Hughes 1989). Isopods are the most researched group for turn alternation patterns (Hughes 1967, 1978). When facing obstacles, isopods turn in opposite directions to create a deviation from linear motion (Hughes 1989) without being forced to do so by other external factors (Dingle 1965). Turn alternation patterns are probably caused by internal reactions to foot movements (Beal and Webster 1971). Several studies regarding this topic have been carried out (Kupfermann 1966; Hughes 1967, 1978, 1985, 1987, 1989, 2008; Moriyama 1999). Turn alternation pattern enables an escape from places with unfavourable conditions, such as lack of food (Hughes 1978), vibrations (Houghtaling and Kight 2006), the presence of predators (Carbines et al. 1992), and dehydration (Hughes 1967). Thus, turn alternations can serve as an indicator of stress. It is known that terrestrial isopods increase turn alternations in unfavourable environmental conditions to escape effectively, but previous habituation to disturbance can significantly reduce the stressor’s effect (Houghtaling and Kight 2006). The extent of stress impact on turn alternation patterns depends on how long the isopods will be exposed to stress factors, i.e., how isopods will accustom or acclimate to a given source of stress (Warburg 1964; Cloudsley-Thompson 1969). Cividini and Montesanto (2018a) investigated the effect of vibrations on the alternate turns of isopods. They observed the increase of turn alternations rate in adult individuals of *Armadilloofficinalis* Dumeril, 1816 with the presence of vibrations when compared to *Armadillidiumvulgare* (Latreille, 1804). The ability to perceive and respond to substrate-transmitted vibrations, in conjunction with alternate turns, increases with age (Cividini and Montesanto 2018b). Animals are likely to interpret species-specific and non-specific substrate-borne stridulations as a source of potential danger (Cividini et al. 2020). Turn alternation as an antipredatory strategy of woodlice has been examined by many authors such as Carbines et al. (1992), Houghtaling and Kight (2006), Hegarty and Kight (2014), and Cividini and Montesanto (2018b).

The formation of aggregations can be considered as an evolutionary successful reaction to unfavourable temperature, water loss, or predator pressure (Broly et al. 2013). One of the main reasons is that the isopods forming the aggregation makes individuals lose less water and are thus much less affected by the lack of humidity of the environment. Another reason is the reduction of CO_2_ production (Allee 1926). Aggregation is mainly affected by thigmotaxis, attraction by individuals of the same species (Devigne et al. 2011), or by negative phototaxis. According to Allee (1926), there are two main types of grouping. True aggregation represents the stacking of individuals’ bodies on top of each other with strong cohesion. More diffuse aggregations are typical by the lower number of individuals, higher mobility, and shorter length of contacts. Cividini and Montesanto (2018c) investigated the effect of vibrations on aggregation rates in *A.officinalis* and *A.vulgare*. Consistent with their previous work (Cividini and Montesanto 2018a), they found that *A.officinalis* responded to vibrations significantly and avoided zones of higher vibration intensity. Their ability to form large aggregations was lowered probably due to a reduced ability to find other individuals. In comparison to a sample of individuals with the absence of vibrations, they formed a large number of small aggregations. Even though aggregation behaviour in woodlice was examined in many works (Broly et al. 2013, 2014; Broly and Deneubourg 2015; Pogson 2016), understanding about the impact of aggregation on predation in terrestrial isopods is still relatively weak.

Despite turn alternations, Cividini and Montesanto (2018c) found that stressing conditions can alternate the aggregation behaviour of some terrestrial isopods too. Thus, the goal of this study was to test this claim on different species of terrestrial isopods and environmental stimuli. We evaluated the level of stress of two species exposed to substrate microvibrations, increased temperature, or their combination using turn-alternation in a T-maze. We assumed that both factors are stressful for isopods. Next, we tried to analyse the level of stress on aggregation behaviour under the same conditions.

## Materials and methods

*Porcellioscaber* Latreille, 1804 (9–14 mm length) were hand-picked in an urban area of the village Bučovice, while *Porcellionidespruinosus* (Brandt, 1833) (3–8 mm length) were collected from a garden compost in the town of Hodonín. Woodlice were placed in 17 × 17 × 8 cm plastic boxes with a thin layer of plaster to maintain humidity, with egg cartons used as an underlay. The plaster was kept moist, and isopods were fed on carrots ad libitum. Animals were kept in constant darkness with a temperature of 18–20 °C.

### The experiment

The behavioural reaction to two stress factors or their combination was observed in both species. The first factor was increased temperature (27–31 °C, treatment coded as T+) while normal temperature (18–24 °C, called lower temperature and coded at T-) was used as a control. The second factor was the presence of microvibrations (coded as V+) and the absence of vibrations served as a control (V-). The experiments were carried out from August to October 2020. Before the beginning of each experiment, woodlice were exposed to a specific combination of conditions (T+V+, T+V-, T-V+, or T-V-, respectively) for two hours.

Two speakers with a power of 5 W were used to test the responses of the isopods to non-specific vibrations. There was a chipboard plate on the top of the speakers. The vibrations were produced by an artificially created recording, the same as the recording used by Cividini and Montesanto (2018a, b, c). The sound was played on an MP3 player connected to the speakers. Vibrations were measured with an oscilloscope application from a mobile phone placed of a T-maze between speakers, using the VibSensor application. RMS values of vibrations during minute measurement were X: (0.03); Y: (0.04); Z: (0.055) m/s^2^.

**Figure 1.**
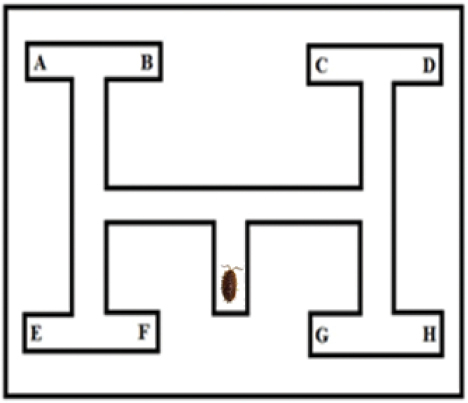
Scheme of T-maze (labyrinth) used for evaluation of turn alternations.

To measure alternating turn behaviour, we used a plastic T-maze of size 15 × 11 cm consisting of two parts, i.e., the bottom labyrinth part and the cover with a small hole that served as an entry spot for subjects. Isopods were placed into the maze with entomological forceps, and then we observed how they alternate turns when trying to reach one of the six possible ending points. To prevent that woodlouse follow conspecific cues left by the previous woodlouse, the plastic labyrinth was placed on white A4 paper which was replaced after each trial. We also recorded changes in isopod´s turn decision (isopod made U-turn and used opposite corridor), and the time needed for reaching one of the end points of the labyrinth (Fig. 1). If woodlice did not start to move within 3 min, trial was terminated. To simplify the calculation of turn alternation pattern, a specific value was assigned to each end point of the labyrinth (depending on how many alternating turns isopod made). Reaching the points A or D with assigned value 1 meant that turn alternation pattern was systematic, i.e., turn left-right-left (L-R-L) or turn right-left-right (R-L-R, see Fig. 1). Points B and C had a value of 1.6 because animals started turn alternation pattern but did not complete it (turns L-R-R, or R-L-L). Ends E and H with a value of 2.3 indicated that isopods did not start with a turn alternation pattern from the beginning but only after the second turn (turns L-L-R, or R-L-L). Reaching the points F and G with the value of 3 was considered the result of not systematic turn alternation (R-R-R or L-L-L).

To observe aggregation behaviour, 30 individuals of the same species were placed into a box, and recorded on camera for two hours. Before the experiment, the plaster inside each box was thoroughly moistened to provide enough humidity. For filming, a small Niceboy outdoor camera installed on a tripod was used. We analysed 12 images (one every 10 min) of each video and calculated the number of isopods touching each other, i.e., the presence of thigmotaxis. After filming, isopods were returned to the breeding boxes. The results are expressed as the average aggregation dynamics for all four variants of observation. In total, 46 aggregation dynamics of *P.scaber* and 49 aggregation dynamics of *P.pruinosus* were analysed.

### Data analysis

For T-maze experiments, we analysed the level of turn alternations according to the end point, the time needed for reaching the end point, and the number of changes in turn alternation. For aggregation experiments, the number of aggregated animals (individuals in contact) every ten min were analysed. All results were evaluated using a one-way ANOVA with a significance level of α = 0.05. The presence of vibrations with the increased temperature was coded by the number 1, while the absence of both factors was marked as 0. Pearson’s correlation test was used to evaluate the dependence of the turn alternation and the speed of passage through a maze.

## Results

### Turn alternation

Three behavioural characteristics of movements were examined in the maze. The first was the rate of a random ramble (negatively correlated with turn alternation). The second variable was the time spent in the labyrinth, measured from the entry of an isopod into the maze until it reached one of the possible end points. The third variable was the extent of changes in turn alternation pattern, i.e., the number of returns and changes in the turn alternation in the labyrinth. A total of 280 individuals of *P.scaber* and 301 individuals of *P.pruinosus* were tested in this type of experiment.

Our results did not show a statistically significant association between the rate of a random/unspecific ramble (reversed value of systematic turn alternation) and the presence of microvibrations (F = 0.09; p = 0.761) for *P.scaber*. The average rate of a random ramble for the presence of vibrations was 1.74 and for the absence of vibrations was 1.71. There was no significant effect of vibrations on the time spent in the labyrinth (F = 1.45; p = 0.229), although the individuals of *P.scaber* exposed to vibrations ran through the labyrinth with an average time of 38 sec vs. 45 sec with the absence of vibrations. Also, there was no significant association between the presence of vibrations and changing of the turn alternation pattern for this species (F = 0.20; p = 0.657). An average number of changes during the presence of vibrations was 0.87 in contrast with 0.77 during their absence.

In contrast, for *P.pruinosus*, the association between the rate of a random ramble and the presence of microvibrations was statistically significant (F = 5.01; p = 0.026). The average rate of a random ramble during the presence of vibrations was 1.86 and during their absence was 1.67. Isopods made more systematic turn alternation with the absence of vibrations. There was no significant effect of vibrations on the length of the time spent in the labyrinth (F = 0.03; p = 0.862). The average time spent in the labyrinth with the presence of vibrations was 37 sec while with the absence of vibrations it was 38 sec. There was no significant association between the presence of vibrations and change of turn alternation (F = 2.67; p = 0.103). An average number of changes in turn alternation with the presence of vibrations was 1.13 and 0.74 when vibrations were absent.

In the case of *P.scaber*, we found out the significant associations between increased temperature and the rate of a random ramble (F = 21.84; p < 0.001). The average rate of random ramble during exposure to increased temperatures was 1.92, while at lower temperatures it was 1.52. Thus, this species made less alternating turns in an increased temperature environment. Results also showed a statistically significant association between the time spent in the labyrinth and increased temperature (F = 30.65; p < 0.001). Individuals exposed to increased temperatures ran through the labyrinth with an average time of 58 seconds while in lower temperatures it was 25 seconds. Thus, isopods spent more time in a maze when temperatures were increased. We also found a significant association between temperatures and changes in turn alternation pattern (F = 25.56; p < 0.001). An average number of changes of turn alternation during exposure to increased temperature was 1.33 in comparison to 0.30 at lower temperature. An increasing number of changes in turn alternation was observed in woodlice behaviour when the temperature was increased.

For *P.pruinosus*, the associations between increased temperature and the rate of a random ramble were not significant (F = 0.02; p = 0.891). The average rate of random ramble during exposure to increased temperatures was 1.76, while at lower temperatures it was 1.77. There was no significant association between the time spent in the labyrinth and increased temperature (F = 0.79; p = 0.375) for this species. The average time spent in the labyrinth was 35 seconds per individuals exposed to increased temperatures and 40 seconds per those exposed to lower temperatures. We prove the existence of a significant association between temperatures and change of turn alternation pattern (F = 4.44; p = 0.036). An average number of changes in turn alternation during exposure to increased temperature was 1.2; for lower temperatures it was 0.7. For *P.pruinosus*, the increased temperature significantly increased the number of changes in turn alternation pattern.

**Figure 2.**
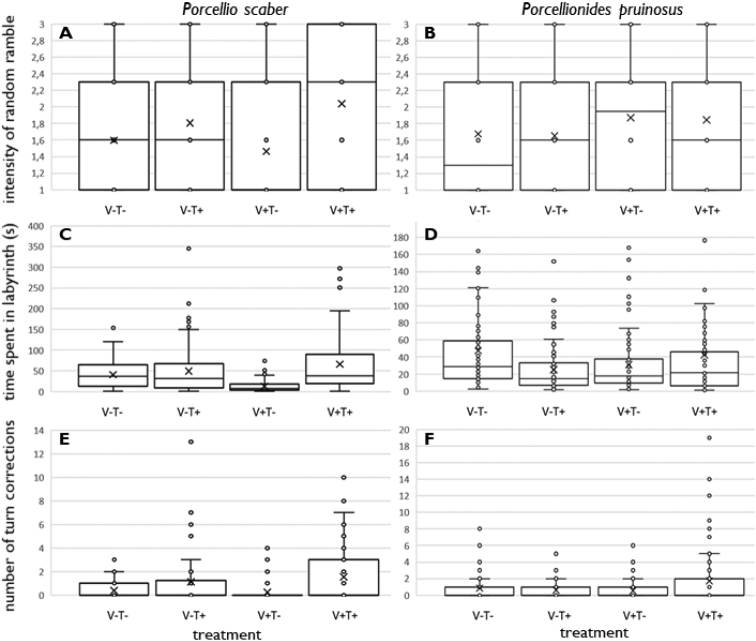
Characteristics of movement in T-maze of *P.scaber* (**a, c, e**) and *P.pruinosus* (**b, d, f**) at different treatments: **a, b** intensity of random/unspecific ramble, i.e., reversed value of systematic turn alternation **c, d** speed of passing through the labyrinth **e, f** number of self-corrective turns. Treatments V-/V+ mean absence/presence of substrate vibrations and T-/T+ mean low/high temperature.

Combination of increased temperatures and the presence of vibrations has significant effect on rate of random ramble (F = 8.99; p < 0.001; Fig. 2a) for *P.scaber*. The highest average rate of the random ramble was at increased temperatures with the presence of vibrations (2.04) in opposite to lower temperatures with the presence of vibrations (1.47). Also, the effect of a combination of temperatures and vibrations on time spent in the labyrinth was statistically significant (F = 15.80; p < 0.001; Fig. 2c). *Porcellioscaber* ran through the labyrinth the fastest at lower temperatures with vibrations (13 sec) and the slowest at increased temperatures associated by vibrations (66 sec). The vibrations thus increased the isopod´s speed while the increased temperature slowed them down. The effect of the combination of increased temperature and vibrations on changes in turn alternation was significant (F = 9.38; p < 0.001; Fig. 2e) for this species. In average, highest numbers of changes in the turn alternation were made at increased temperatures associated by vibrations (1.55 changes) compared to lower temperatures with presence of vibrations (0.24 changes). The presence of vibrations thus reduced the number of changes in turn alternation, while the higher temperature, on the contrary, increased them.

For case of *P.pruinosus*, combination of increased temperatures together with presence of vibrations has no significant effect on rate of random ramble (F = 1.69; p = 0.170; Fig. 2b). The highest average rate of the random ramble was at low temperatures along with the presence of vibrations (1.87) and the lowest at increased temperatures with the absence of vibrations (1.65). The effect of a combination of temperatures and vibrations on time spent in the labyrinth was statistically significant (F = 3.34; p = 0.020; Fig. 2d). Isopods ran through the labyrinth with the highest speed at increased temperatures with the absence of vibrations (25 sec) and the slowest at lower temperatures with the absence of vibrations (47 sec). We proved the existence of statistically significant effect of the combination of increased temperature, vibrations on the change of turn alternation (F = 5.38; p = 0.001, Fig. 2f). On average, highest numbers of changes in the turn alternation were made at increased temperatures combined with the presence of vibrations (1.71) and the lowest numbers when isopods were exposed to lower temperatures combined with the presence of vibrations (0.54).

We also found out a significant weak positive correlation (R = 0.32, p < 0.00001.) between the intensity of the turn alternation of *P.scaber* (i.e., the probability of running to one of the ends, indicating systematic turn alternation like A or D) and the speed of passage through the maze. When *P.scaber* ran slowly, there was higher probability that it will reach one of the “wrong” ends, which indicates unsystematic alternating turns. Results for *P.pruinosus* show no correlation (R = 0.06, p = 0.264619) between the intensity of the turn alternation and the speed rate of passage through a maze.

### Aggregation

Group of two or more woodlice in contact were considered to be an aggregate. The distributions of the individuals were determined by counting the number of aggregated individuals in each box every 10 min during the 120-min experiment.

**Figure 3.**
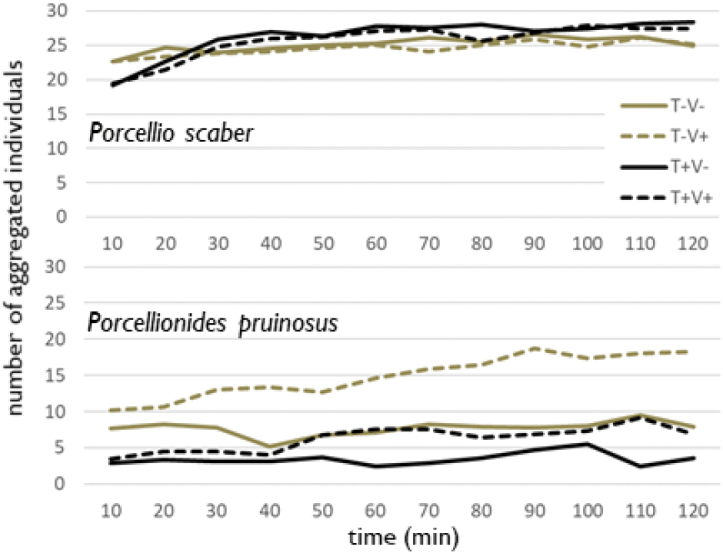
Dynamics of aggregations of *P.scaber* and *P.pruinosus* during 2-hr observations at different treatments. V-/V+ mean absence/presence of substrate vibrations and T-/T+ mean low/high temperature.

For *P.scaber* the results showed that there is a statistically significant difference in aggregation dynamics of isopods exposed to vibrations (F = 5.71; p = 0.003). Fig. 3 shows that the isopods presented different aggregation dynamics depending on different temperatures. At a lower temperature, ~ 23–26 individuals (out of a total number of 30) were aggregated during the whole two hours of observation (Fig. 3). In comparison, at increased temperatures the aggregations were initially smaller (20 individuals) but increased within half an hour, and stabilised at the number ~ 25–28 individuals (Fig. 3). Aggregations on a vibrating surface were usually smaller than aggregations on a stable substrate.

In *P.pruinosus*, vibrations together with increased temperature had a significant effect on the dynamic and size of aggregation (F = 83.52; p < 0.001). A higher number of aggregated individuals was observed among isopods exposed to lower temperatures combined with the presence of vibrations (after an hour, half of the total of 30 individuals were in aggregations). In comparison, numbers of aggregated isopods were the lowest at increased temperature with the absence of vibrations. In all of the observed variants it can be seen a slight increase in the number of aggregated individuals over time (Fig. 3). Aggregations tended to repeatedly appear and disappear.

## Discussion

### Turn alternation

Our results showed that the effect of vibrations on the rate of turn alternation of *P.scaber* was not significant. When the substrate did not vibrate, *P.pruinosus* significantly increased turn alternations. For both species, the vibrations did not affect the time spent in the labyrinth or changed turn alternation pattern. This is probably because neither *P.scaber* nor *P.pruinosus* has any stridulatory or auditory organs to absorb vibrations. Those can be found in species like *A.officinalis* that is probably able to generate and receive vibrations (Cividini and Montesanto 2018a, 2020). The presence of mechanoreceptors that detect substrate vibrations in isopods is well documented (Zimmerman and Kight 2016), but the monotonous vibrations probably do not resemble an oncoming predator. The effect of monotonous and interrupted substrate vibrations on isopod behaviour should be studied in future studies.

A different effect of vibrations to turn alternations was reported by Moriyama et al. (2016), who observed woodlice at room temperature. In their study, approximately a quarter of the woodlice exposed to vibrations alternated their directions. Houghtaling and Kight (2006) proved that habituation to disturbance can significantly reduce turn alternations. Insignificant reaction to vibrations could be caused by the fact that before each experiment, woodlice were exposed to vibrations for a period of two hours. The animals could be used to the vibrations, thus the weak response during the experiment.

An increased temperature did not stress *P.scaber*, because isopods spent more time in the maze and alternated their turns less systematically. This result is unexpected because it is contrary to the expectation based on the findings of Warburg (1964) and Schuler et al. (2011), who reported that at increased temperatures, *P.scaber* ran faster. It is also contradictory to the findings of Cloudsley-Thompson (1956), who mentioned that increased temperatures represent a stressful factor for woodlice, mainly due to its impact on water loss (causing their gradual drying and therefore triggering more movement). In contrast, there was no significant effect of increased temperature on the rate of a random ramble or on the time needed to complete the labyrinth in *P.pruinosus*. This species can be commonly found in compost or stacked bales of hay (Frankenberger 1959), which means they could be used to higher temperatures that compost goes through during intense microbial decomposition. *Porcellionidespruinosus* is more tolerant to increased temperatures showing a stable feeding rate for 20 °C as well as 28 °C than *P.scaber* is (Römbke et al. 2011). Meanwhile, *P.scaber* collected in Central Europe has a temperature optimum of 21 °C (Antol et al. 2019), indicating that increased temperature should be considered a stress factor for this species. After exposure to increased temperatures, both species made a higher number of returns or turn alternations. Hughes (1967) reported, that alternation of turns can be caused by the effort to escape from adverse conditions. Moriyama et al. (2016) found that ca. a quarter of the total number of tested woodlice made more returns or changes in the turn alternation pattern. The effect of increased temperatures was also mentioned by Warburg (1964), who found out that the *Oniscusasellus* Linnaeus, 1758 as well as *A.vulgare*, made more alternating turns after the exposure to increased temperatures. In our experiment, *P.scaber* ran slower and alternated turns less systematically at increased temperatures. This could be caused by the fact that before each experiment, isopods were exposed to the tested temperature for two hours. Khan and Khan (2008) reported loss of body mass of the water flea *Daphniamagna* Straus, 1820 in increased temperature resulting from previous hyperactivity. In similar fashion, *P.scaber* could be exhausted and therefore did not show a stronger activity. Also, Ferreira et al. (2016) stated that *P.pruinosus* shows signs of stress when exposed to temperatures above 30 °C, while to the temperatures below 20 °C it does not react at all. Refinetti (1984) and Nair et al. (1989) reported that *A.vulgare* and *Porcelliolaevis* Latreille, 1804 can quickly acclimatise to increased temperature. Nevertheless, this may not apply for *P.scaber* from Central Europe, as both mentioned papers deal with (sub)tropical populations.

Vibrations, together with increased temperature, have a significant effect on the rate of random ramble, time spent in the labyrinth, as well as the change of turn alternation in *P.scaber*. These results have the same pattern as those with increased temperature alone. Apparently, vibrations were not stressful for *P.scaber*, probably due to its origin in the city environment. Houghtaling and Kight (2006) reported that urban isopods were adapted to microvibrations. Our results showed that the combination of temperature and vibrations, similar to increased temperature alone, did not affect turn alternation of *P.pruinosus*, potentially due to characteristics of the places they typically occur at. The increased temperature together with vibrations significantly affected changes in turn alternation. Changes in turn alternation were significantly affected by the higher temperature together with vibrations, as well as by the higher temperature alone.

### Aggregations

*Porcellioscaber* showed a statistically significant difference in aggregation dynamics after their exposure to vibrations. At lower temperatures, somewhat stable aggregations of ~ 23–26 individuals were formed. At increased temperatures, within half an hour, the number of aggregated isopods increased to approximately 28 individuals and then stabilised. This is probably because the optimal temperature for *P.scaber* is 21 °C (Antol et al. 2019) (i.e., our experimental “lower temperature”), and because at colder conditions water loss does not occur as quickly as at increased ones (Cloudsley-Thomspon 1956). Woodlice exposed to increased temperatures tend to lose water faster, so they start to aggregate to prevent water loss. Isopods that are in the upper layer of crowded individuals leave the group more often to look for a more suitable place due to the quicker water loss (Allee 1926).

The aggregation of *P.pruinosus* was significantly affected by vibrations along with increased temperatures. Isopods aggregated more when exposed to lower temperatures. In an hour after the exposure more than half of the individuals were aggregated. This is in agreement with results of Cividini and Montesanto (2018c) regarding *A.officinalis*. This is probably related to the finding of Allee (1926), who mentioned that woodlice form two types of aggregations, namely the bunching or true aggregation that is seen in *P.scaber* and crowding or a more diffuse grouping observed in *P.pruinosus*, depending on their mutual contact and interactions. The isopods aggregated less at increased temperatures than at the lower one. According to the findings of Allee (1926), when isopods were under conditions unfavourable to aggregation such as the exposure to low temperatures, the tendency to aggregate increased. However, the increased temperature was not a stressful factor for *P.pruinosus*, probably because this species is used to the increased temperatures. Numbers of aggregated isopods were slightly higher due to vibrations.

*Porcellionidespruinosus* aggregated in greater numbers during the presence of vibrations than during non-vibration treatment. The same results showed an experiment by Cividini and Montesanto (2018c), who tested the effect of vibrations on *A.officinalis* at room temperature (20 °C). In the non-vibration treatment, isopods quickly began to aggregate and formed a single stable aggregation. However, *A.officinalis* aggregated less with the absence of vibrations. Cividini and Montesanto (2018c) mentioned that isopods could create a sound by rubbing different parts of the body against each other when conglobated, which could evoke other individuals to stay inactive and do not aggregate. Although *P.pruinosus* is not able to stridulate, its antipredatory response is not volvation, only running away and looking for hiding place.

Based on our findings, the pairing model species – stressor can be further refined for more significant results. Further research should be aimed at how stressful various temperature ranges for different isopod species are. Due to the ability of woodlice to acclimate to substrate vibrations, the future use of vibrations in experimental studies is very problematic. Perhaps shorter experiments with low air humidity as a stressor can be less difficult as our knowledge about the demands of different species is sufficient. The ability of different isopod species to habituate to stress factors could also play a certain role in this matter and future studies of this topic are encouraged.

## Conclusions

Our results showed that for *P.scaber* and *P.pruinosus*, vibrations are not a stressful factor. This may be related to the fact that tested individuals have been collected in an urban environment where road and rail transport is a permanent source of substrate microvibrations, and the isopods are used to it. The increased temperature was a stressor only for *P.scaber*. This species did not show any major response to increased temperature; it went through the labyrinth more slowly at increased temperatures, and although they made more changes, they alternated turns less intensely. This behaviour could be caused by previous too long exposition to experimental conditions. *Porcellionidespruinosus* was not stressed by the increased temperature, which is probably caused by their occurrence in composts, where the temperature is often increased due to intense microbial decomposition. The aggregation dynamics of *P.scaber* was affected by the increased temperature. Initially, at increased temperatures isopods aggregated less or formed more unstable aggregations than the control group, but later the aggregations were stable and slightly larger than in the control group. Thus, the aggregation behaviour of *P.scaber* shows certain degree of stress, but its interpretation is relatively complicated. We were not able to confirm that *P.pruinosus* was stressed by vibrations or temperature, so it was not possible to make the comparison of the aggregation behaviour and the degree of stress. These results suggest that factors that have been used as stressors for specific species in some studies cannot be automatically used as stressors for other terrestrial isopod species.
